# Feature Selection Model based on EEG Signals for Assessing the Cognitive Workload in Drivers

**DOI:** 10.3390/s20205881

**Published:** 2020-10-17

**Authors:** Patricia Becerra-Sánchez, Angelica Reyes-Munoz, Antonio Guerrero-Ibañez

**Affiliations:** 1 Department of Computer Architecture, Polytechnic University of Catalonia, 08034 Catalonia, Spain; angelica.reyes@upc.edu; 2 Telecommunications Department, University of Colima, 28040 Colima, Mexico; antonio_guerrero@ucol.mx

**Keywords:** electroencephalographic, feature selection, machine learning, prediction model

## Abstract

In recent years, research has focused on generating mechanisms to assess the levels of subjects’ cognitive workload when performing various activities that demand high concentration levels, such as driving a vehicle. These mechanisms have implemented several tools for analyzing the cognitive workload, and electroencephalographic (EEG) signals have been most frequently used due to their high precision. However, one of the main challenges in implementing the EEG signals is finding appropriate information for identifying cognitive states. Here, we present a new feature selection model for pattern recognition using information from EEG signals based on machine learning techniques called GALoRIS. GALoRIS combines Genetic Algorithms and Logistic Regression to create a new fitness function that identifies and selects the critical EEG features that contribute to recognizing high and low cognitive workloads and structures a new dataset capable of optimizing the model’s predictive process. We found that GALoRIS identifies data related to high and low cognitive workloads of subjects while driving a vehicle using information extracted from multiple EEG signals, reducing the original dataset by more than 50% and maximizing the model’s predictive capacity, achieving a precision rate greater than 90%.

## 1. Introduction

Driving a vehicle is a complex activity exposed to demands that continually change due to different factors, such as the speed limit, obstacles on the road, and traffic, among others. When performing this activity, drivers must have a high degree of concentration, increasing the demand related to the cognitive workload, or cause vehicle accidents due to carelessness [1]. In recent years, various tools have been used to assess the demand for the cognitive workload generated in drivers, such as subjective measures [2,3], vehicle performance measures [4,5], and physiological measures [6,7], with electroencephalographic (EEG) signals having been the most frequently used to identify cognitive states due to their high precision [8].

EEG signals allow the behavior of a person’s brain activity to be analyzed in real-time. However, this type of physiological signal generates a lot of information per second, which increases proportionally according to the collection time and the number of sensor channels, consequently producing large volumes of data and resulting in complex and robust treatment [9,10].

One of the main challenges facing EEG signals is finding the right information for identifying cognitive states. Considering this, feature selection methods have been developed for pattern recognition using physiological signals. The feature selection algorithms (FS) aim to find a set of features with relevant information or data that can identify or describe an event, allowing the performance of the prediction models to be maximized [11].

Many investigations have developed models implementing FS to identify the cognitive workload using the physiological signal’s information. In [12], it is shown that soft computing-based EEG classification by extracting and then selecting optimal features can produce better results. The system displays an accuracy of 93.05% and 85.00%, obtaining a low performance in real-time environments. In [13], an attention-based convolutional recurrent neural network (ACRNN) is presented to extract features from EEG signals and improve the emotion recognition accuracy. The system achieves average accuracies of 93.72% and 97.73% and improves the emotion recognition accuracy by approximately 2% and 1%. In [14], an effective multi-level feature guided capsule network is proposed to extract characteristics from the EEG signals and determine the emotional states. The method achieves an average accuracy of 97.97% and 94.59% and presents network complexity. In [15], a channel selection method is presented to select an optimal subset of EEG channels using normalized mutual information (NMI). The system achieves a 74.41% and 73.64% accuracy and the channel selection method slightly improves the recognition rate. In [16], a system for selecting and classifying EEG signals based on common spatial patterns (CSP) is proposed, obtaining an 84.8% accuracy. The system does not include a parameter regularization method and does not consider a real-time environment. In [17], eight different machine learning and feature selection algorithms are used to reduce the number of features and improve the classification performance, achieving a 97.74% accuracy. Some algorithms slightly reduce its performance after feature reduction. In [18], a system for selecting and classifying mental stress that implements statistical techniques and SVM, Naive Bayes, and Multilayer Perceptron is proposed, achieving a 92.85% accuracy. This system uses information from a small dataset. In [19], an emotion recognition system for affective states is developed based on the EEG signal using a support vector machine (SVM) classifier. The classifier obtains a 75% and 71.21% performance accuracy and presents problems associated with identifying negative emotions. In [20], the authors present a quaternion-based signal analysis technique based on EEG signals to extract the registered cognitive activity features. The model achieves an 86.44% accuracy and requires a minimum limit of samples to obtain better results, increasing the analysis and information processing time. In [21], an on-line classification method based on common spatial patterns is presented for feature extraction, using SVM as a classifier and achieving an 86.3%, 91.8%, and 92.0% accuracy. In [22], different classifiers are developed using linear discriminant analysis, quadratic discriminant analysis, k-nearest neighbor, SVM linear, the SVM radial basis function (RBF), and naive Bayesian based on EEG signals. SVM obtains the best accuracy of 82.14%. In these systems, the strategy employed to extract the information can cause a loss of vital data. In [23], the authors propose a system for detecting vigilance levels using EEG signals and combine SVM algorithms with multi-particle optimization, obtaining an 84.1% accuracy. The model displays a low prediction performance in some predictions due to the complexity of the data. In [24], the authors develop a model for predicting the mental workload based on a linear discrimination function, achieving an 85% accuracy. In this model, some physiological measures cannot effectively reflect the mental workload, affecting the model’s prediction precision. In [25], the common spatial pattern algorithm is used to extract information from EEG signals and a classifier is developed using the extreme learning algorithm, obtaining an 87.5% accuracy. The model presents a high sensitivity to the kernel configuration, affecting its performance. In [11], the evolutionary computing algorithm is used to find an optimal dataset, obtaining a 96.97% accuracy. This model presents premature convergence problems in the evolutionary algorithm. Finally, in [26], the Bayesian low learning algorithm is implemented to select a dataset. SVM with RBF is employed, achieving an 89.7% accuracy. This model presents problems in the data collection phase.

In summary, several models have been proposed for pattern recognition in recent years using FS algorithms based on physiological signals. The models described above have been based on traditional techniques that reduce the minimum percentage of the original dataset features, obtaining robust prediction models or analyzing the information from a specific signal to compare several features using small datasets and discarding relevant information. Here, we propose a new feature selection model for pattern recognition using information from EEG signals called Genetic Algorithms and Logistic Regression for the Structuring of Information (GALoRIS). GALoRIS combines genetic algorithms (GAs) and logistic regression (LoR), to create a new fitness function and explore the fusion of EEG information, identifying the critical features that contribute to recognizing cognitive states, optimizing the classification process. The dataset obtained from applying the FS algorithm is used as the index for recognizing cognitive states in the predictive model.

GAs are adaptive and robust computational procedures based on the mechanism of natural genetic systems inspired by the natural evolution theory of Charles Darwin [27]. GA is used to solve a complex model’s optimization problems, by looking for the best feature set, especially when the search space is large and complex [18].

Traditional GAs are based on the evolutionary process, which consists of selecting and combining different characteristics, evaluating each dataset to obtain a set that best adjusts the resolution of the problem [28]. The evolutionary process is performed in parallel in multiple directions, creating large populations, ruling out possible solutions, and generating computationally expensive models [29].

To address this problem, we propose a new fitness function based on the LoR classifier’s performance, in order to guide the GA search direction towards the optimal solution. LoR is a technique characterized by its effectiveness, simplicity, and use of a low computational resource. LoR models the probability of each element selected by GA, obtaining the feature’s weight to evaluate its level of competence with the rest of the possible solutions, eliminating multidirectional searches in parallel and storing the best features to create new and better populations.

The results obtained from the GALoRIS model are implemented as indexes of EEG signals for pattern recognition in four classifiers developed with SVM with a linear kernel and RBF, linear regression (LiR), and k-nearest neighbors (k-NN) and predict two cognitive states: A low and high cognitive workload.

The main contributions of this paper can be summarized as follows: (Section 2.1) A new collection criterion method based on statistical techniques is employed to construct an EEG dataset defined as a search space that GALoRIS uses to explore the information; (Section 2.2) the new feature selection model called GALoRIS is presented; (Section 2.3) a new chromosomal structure is defined to direct the search for the features. (Section 2.4); a new fitness function is proposed based on the LoR classifier’s performance to determine the search direction of GA; (Section 2.5) a new technique for chromosome selection is proposed; (Section 2.9) an original method is employed to structure the information of multiple EEG channels, supporting datasets of various sizes; (Section 2.11) a new methodology is presented for labeling the data that calculates the interval ranges of the information to evaluate the subject’s cognitive states.

The rest of the work is organized as follows: Section 2 describes the methodology; Section 3 presents the experimentation implemented; the results are presented in Section 4; and finally, the conclusions and discussions are given in Section 5.

## 2. Methodology

In this investigation, EEG signal information was collected from subjects while they faced a real driving scenario. Additionally, information on subjective measures (NASA-Task Load Index (TLX) and Instantaneous Self-Assessment (ISA)) and vehicle performance measures (error rate (ER)) were collected to evaluate the cognitive states of the subject during the experiment.

EEG signals were processed using the power spectral density (PSD) to extract the most representative features in the context of the cognitive workload. These characteristics were defined as frequency bands: Delta (0.5–4 Hz); Theta (4–8 Hz); Alpha (8–12 Hz); Beta (12–30 Hz); and Gamma (30–100 Hz) [30]. 

To analyze the collected information, Student’s t-test was used to identify statistically significant differences in the data collected during the experiment and establish a collection criterion to discard information, in order to construct a new dataset defined as a search space that GALoRIS uses to explore the data. Pearson’s correlation coefficient was also implemented to identify the association between ISA, NASA-TLX, and ER and the EEG signal, in order to assess whether the subject experienced an internal cognitive workload during the different phases of the experiment [31]. 

GALoRIS was developed to recognize the most representative features that identify the subject’s low and high cognitive workload states while driving. GALoRIS selects and evaluates the features, identifying the key elements that contribute to recognizing cognitive states and restructuring a new dataset that is implemented in four classifiers developed with the supervised algorithms: SVMRBF, SVMLinear, k-NN, and RiL. 

**Figure 1:**
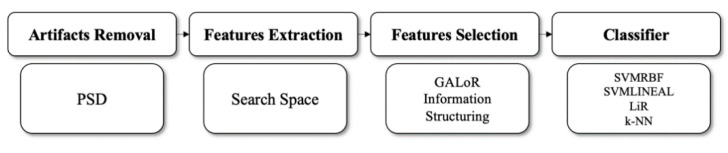
The general architecture of the vehicle driver’s low and high cognitive workload state prediction model.

The general architecture of the cognitive workload prediction model is shown in Figure 1.

### 2.1. Statistical Analysis

Student’s *t*-test was conducted for ISA, NASA-TLX, ER, and the EEG frequency bands signaled the *p*-values where each measure was contrasted with two cognitive workload states. The established hypotheses were

*H*_0_*<*p, there is no significant difference between the information obtained during the two experiments, and

*H*_1_*>*p, there is a significant difference between the information obtained during the two experiments,

where, if the value of the error probability (p) of the samples is greater than the significance level of α=0.05, the hypothesis established in $H_{1}$ is rejected. 

Student’s t-test results of the EEG signals were used to establish an EEG information collection criterion to construct the search space with relevant information that GALoRIS will use to explore the EEG signal’s information. The criterion can be defined as

$$p_{EEG}\leq \alpha ∴search\;space,$$

where samples of EEG with a value of p≤α are set within the search space.

Additionally, as in [32,33,34,35], Pearson’s correlation between the implemented measures was used to determine the association between measures and cognitive states as a validation method for the subject’s internal state. A hypothesis was defined, where, if the EEG signals were correlated with the subjective and vehicle performance measures, the subject experienced the same level of cognitive workload internally and externally. 

Pearson’s correlation coefficient identifies one variable’s relation by calculating an index that measures the degree of connection between the variables. It was applied between the ISA, NASA-TLX, TE, delta, theta, alpha, beta, and gamma measurements. The analysis was performed by correlating the average of the value obtained from each session per measurement (8 measures * 2 tasks) where, if the correlation range was 0, there was no correlation, and if it was −1 or +1, there was a perfect correlation [36].

### 2.2. GALoRIS

**Figure 2:**
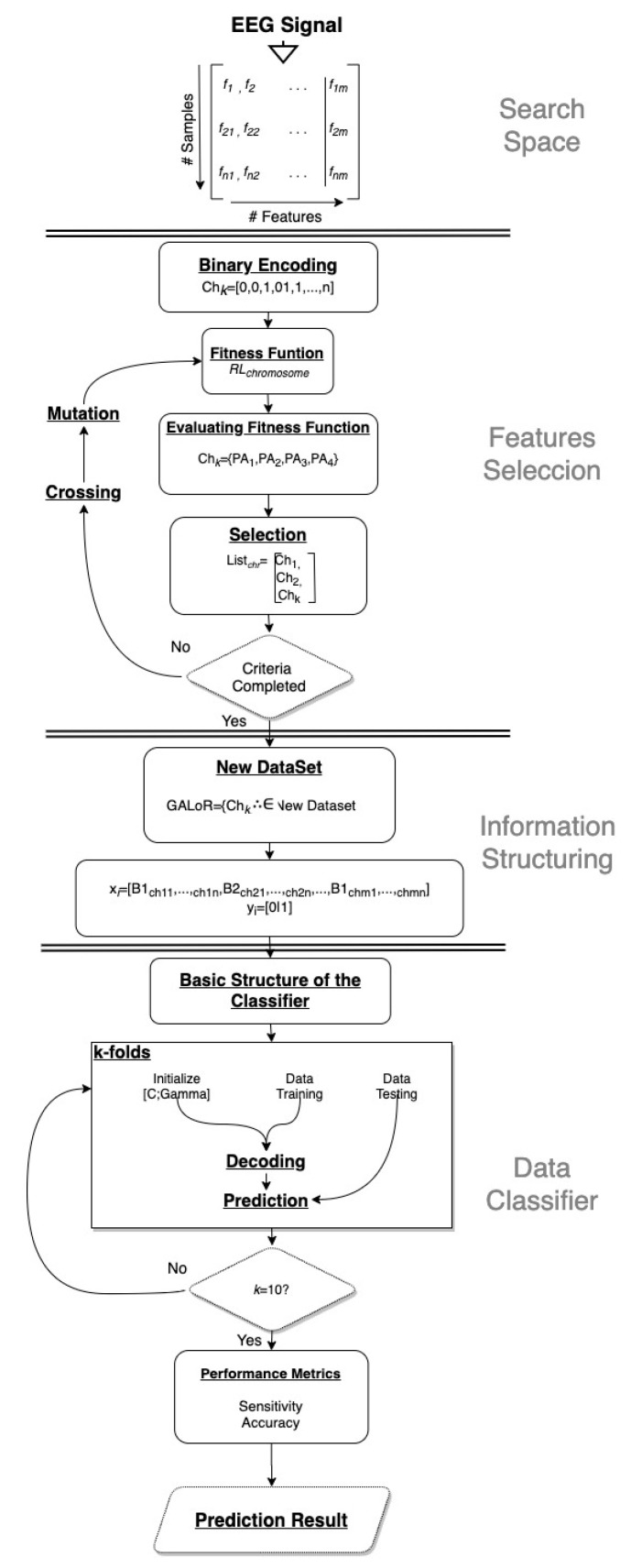
Genetic Algorithms and Logistic Regression for the Structuring of Information (GALoRIS) model architecture for pattern recognition based on the genetic algorithm and logistic regression.

In this section, the architecture of the GALoRIS model is presented. As shown in Figure 2, GALoRIS proposes a new design for the chromosome’s structure and the fitness function based on LoR to model the feature’s weight and determine the direction of the search. Moreover, GALoRIS implements a new selection technique for efficiently identifying the best dataset of features. The model consists of six phases, and they are presented below.

### 2.3. Population

The population is a set defined as an individual or chromosome that represents a possible solution to the problem. The chromosome comprises elements known as genes that are first instantly selected at random. Then, they are modeled through the fitness function. 

A matrix defined as a feature space is built to create the chromosomes, where each element of the matrix presents a gene that the algorithm selects to build a chromosome. The search space is defined as presented in Equation (1):

(1) $$SearchSpace=\left[Delta_{ch_{1\ldots n}},Theta_{ch_{1\ldots n}},Beta_{ch_{1\ldots n}},Gamma_{ch_{1\ldots n}}\right],$$

where the channels of the delta band are organized first, followed by the channels of the theta, alpha, beta, and gamma bands, following the frequency range order. $ch_{n}$ represents the channels of each band, defined as presented in Equation (2):

(2) $$ch_{n}=\left[AF3,AF4,F3,F7,F8,FC5,O2,P8,T8\right],$$

where $ch_{n}$ must meet the collection criterion $P_{EEG}\leq a∴\;\in Search\;Space$. These channels are the key areas of the brain for detecting driving fatigue [37]. The dataset format for the search space is frequency bands ×  channel ×  sample number (5 ×  9 × 8210). All the information is standardized in a range of {0, 1}.

**Figure 3:**
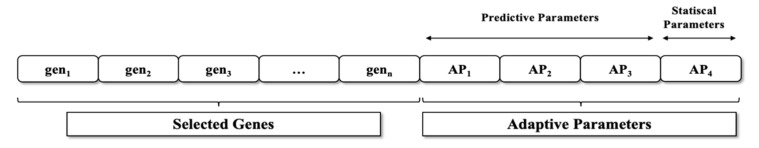
Chromosome structure is built with the information of the selected genes and the weight of each element.

Furthermore, a new chromosome’s structure is defined. The structure contains the features and parameters evaluated for the chromosome to direct the search of elements. The general form of the structure is presented in Figure 3.

Here, $gen_{n}$ represents the chromosome genes encoded in a binary chain gen∈{0,1} i=1,2, …, n; whenever the gene’s value is 1, the feature is selected to form the new chromosome and continue the evolutionary process. $AP_{n}$ represents the adaptation parameters used as evaluation criteria to determine whether the chromosome continues in the evolutionary process.

### 2.4. Fitness Function

The fitness function (FF) evaluates each proposed chromosome’s quality to find the best combination of genes while maintaining a high genetic diversity in the population. The FF calculated for each chromosome generates the adaptation parameters (AP) based on the logistic regression algorithm’s performance. The parameters explore the chromosome’s properties to determine its ability to compete with other chromosomes. The chromosome’s features are divided into two sets. The first set builds the LoR model, and the second set is used to assess the quality of the chromosome and explore the effectiveness of the features according to the AP criteria. Equation (3) presents the general logistic regression model employed to calculate the AP:

$$logist_{AP}=\frac{1}{1+e^{{\left(Chromosome\right)}^{,}}}$$

where

(3) $$Chromosome=\left(\beta_{0}+\sum_{i=1}^{n}\beta_{i}Ban_{chan}\right),$$

where$\;\beta_{0}$ is the intercept, $Ban_{chan}$ represents the chromosome’s selected channels, and $\beta_{i}$ is the estimation coefficient calculated with the logit function for each variable $Ban_{chan}$. It determines the importance of the information provided by each feature based on the global fit of the generated chromosome. In Equation (4), the general chromosome model is presented, implementing all the elements of the search space:

(4) $$\begin{array}{l} chromosome=0.0165+(-97.489)\times T_{AF3}+(-0.969)\times T_{AF4}+1.407\times T_{F3}+3.668\times T_{F7}+0.484\times \\ T_{F8}+(-5.119)\times T_{FC5}+(-1.347)\times T_{O2}+(0.688)\times T_{P8}+0.738\times T_{T8}+(0.298)\times D_{AF3}+0.764\times \\ D_{AF4}+(-1.872)\times D_{F3}+2.429\times \;D_{F7}+(-3.934)\times D_{F8}+4.361\times D_{FC5}+2.538\times D_{O2}+(-1.247)\times D_{P8}\\ +(-1.720)\times D_{T8}+(-1.652)\times A_{AF3}+1.560\times A_{AF4}+2.203\times A_{F3}+(-0.832)\times A_{F7}+1.481\times A_{F8}+(-2.270)\times \\ A_{FC5}+1.806\times A_{02}+1.349\times A_{P8}+2.319\times A_{T8}+(-0.165)\times B_{AF3}+0.855\times B_{AF4}+1.550\times B_{F3}+1.393\times \\ B_{F7}+(-1.542)\times B_{F8}+(-6.378)\times B_{FC5}+2.311\times B_{O2}+2.245\times \;B_{P8}+1.114\times B_{T8}+(-0.964)\times G_{AF3}+0.133\times \\ G_{AF4}+(-0.076)\times G_{F3}+0.274\times G_{F7}+(-0.565)\times G_{F8}+1.571\times G_{FC5}+0.053\times G_{O2}+(-0.079)\times G_{P8}+(-0.377)\times \\ G_{T8}, \end{array}$$

where $\beta_{0}$ and $\beta_{i}$ are estimated from each frequency band ($Ban_{chan}$) and represent the global fit of the search space, where $\beta_{i}$ provides the basis for the feature importance score and calculates each AP. The configuration of the chromosome changes as its elements change.

AP are calculated from the chromosome generated and they are the accuracy of the adjustment of the elements of the chromosome, the error rate for the adjustment, the number of genes of the chromosome, and the significant elements of each chromosome. The parameters are explained below.

The accuracy of the adjustment of the elements evaluates the performance of the generated chromosome and is calculated as presented in Equation (5):

(5) $$\frac{fit\;calculated\;}{{fit\;calculated\;+fit\;incorrectly\;}^{’}}$$

where the number of correctly predicted values divided by the total number is evaluated. The range of values is [0, 1], where 1 indicates a high level of accuracy. 

The error rate for the adjustment of the elements quantifies the error that occurs when predicting each chromosome, evaluating the number of predictions made incorrectly. It is calculated as presented in Equation (6):

(6) TR=Y−Y’,

where the differences between the actual values Y and the predicted values Y’ are calculated. The range of values is [0, 1], where values close to 0 indicate that the chromosome obtained a lower error fit.

The number of genes on the chromosome is used to evaluate the number of selected elements to build the chromosome. This parameter aims to obtain a chromosome with fewer components capable of describing the data’s behavior, reducing the probability of error, analysis time, and algorithm execution.

The significant element evaluates each of the chromosome gene’s contributions by comparing the gene’s *p*-value with the significance level of α=0.05. If the p-value is less than or equal to the significance level, the evaluated variable is relevant and should remain on the final chromosome.

### 2.5. Selection

The selection process consists of building a list of chromosomes using the criteria established in the AP, as described in Equation (7). This process begins by comparing the AP values of each chromosome, where the chromosome with a higher adjustment rate and a lower error rate is positioned at the top of the list. If these parameter values match, the chromosome with the fewest elements will have the highest priority.

(7) $$\begin{array}{c} Chr_{11}=\left\{x_{ban_{11}},x_{ban_{12}},\ldots ,x_{ban_{1n}},x_{AP_{Acc}},x_{AP_{ET}},x_{AP_{NG}},x_{AP_{SE}}\right\}\\ Chr_{12}=\left\{x_{ban_{21}},x_{ban_{22}},\ldots ,x_{ban_{2n}},x_{AP_{Acc}},x_{AP_{ET}},x_{AP_{NG}},x_{AP_{SE}}\right\}\\ Chr_{1m}=\left\{x_{ban_{m1}},x_{ban_{m2}},\ldots ,x_{ban_{mn}},x_{AP_{Acc}},x_{AP_{ET}},x_{AP_{NG}},x_{AP_{SE}}\right\},\;\\ \vdots \\ \mathrm{where}\;Chr_{1m\left[x_{AP_{Acc}},x_{AP_{ET}},x_{AP_{NG}}\right]}>Chr_{1n\left[x_{AP_{Acc}},x_{AP_{ET}},x_{AP_{NG}}\right]}∴highest\;priority\;list \end{array}$$

**Figure 4:**
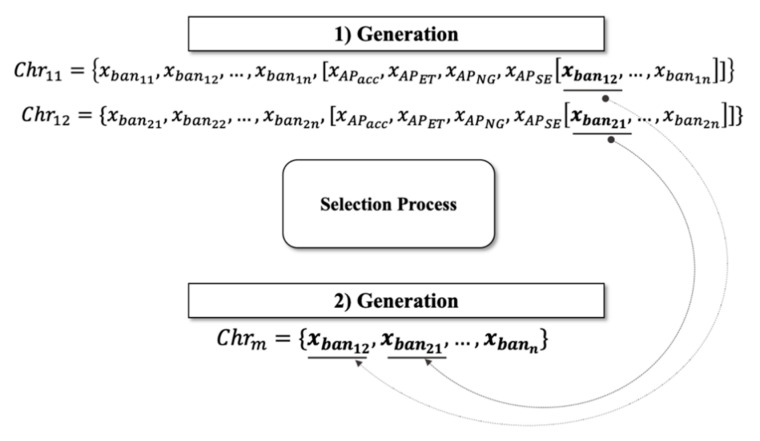
Element selection system used to build new chromosomes with better qualities.

The elements with a value of $x_{ban_{nn}}<a$ are united in the same vector to create a new chromosome and inherited in the next generation, as shown in Figure 4. This process directs the selection of elements to form new chromosomes with better properties, selecting features with relevant information.

### 2.6. Crossing

Once the best chromosomes are selected based on FF, the reproduction process begins with the crossing between chromosomes, as observed in Equation (8). This phase consists of cutting the chromosome at two selected points to generate new segments. One parent’s central segments and the other parent’s lateral segment are chosen to create the descending chromosomes [38]. The crossing provides the possibility of combining all of the chromosome parts to generate chromosomes that are not created in the initial population.

(8) $$\left[\begin{array}{c} Chr_{1}\\ Chr_{2}\\ \begin{array}{c} \begin{array}{c} Chr_{3}\\ \begin{array}{c} Chr_{4}\\ \vdots \end{array} \end{array}\\ Chr_{n} \end{array} \end{array}\right]\begin{array}{c} \begin{array}{c} \begin{array}{c} \to \\ \to \end{array} \end{array} \end{array}\begin{array}{c} Chr_{1}\\ \left[0010101\right]\;\; \end{array}\begin{array}{c} Chr_{2}\\ \left[1010101\right] \end{array}\begin{array}{c} \begin{array}{cc} \to & Chr_{child_{1}} \end{array}\\ \begin{array}{cc} \;\;\;\to & \left[0010101\right] \end{array} \end{array}\begin{array}{c} Chr_{child_{2}}\\ \left[1010101\right] \end{array}$$

### 2.7. Mutation

The mutation generates a new chromosome different from those of the parents to maintain diversity within the population and avoid premature convergence. It consists of randomly inverting part of a gene on the chromosome to obtain variability within the population and discard chromosomes from the new population [38].

### 2.8. Detection Rules

Two stop rules are defined to stop the evolutionary process of the model, of which at least one must be met. The first rule is met when the number of established chromosome generations is completed. This number is defined based on experimentation and the number of features within the search space. The second rule is met when the fitness function’s evaluation criteria are fulfilled (accuracy = 1, error rate = 0).

### 2.9. Information Structuring

A new dataset is constructed based on the feature selection results, integrating the generated chromosome elements to implement it as an input index, in order to recognize patterns in the prediction model. In Equation (9), the general structure employed to build the new dataset is presented:

(9) GALoRIS={chromosome}∴∈New dataset,

where the chromosome represents the new dataset defined as $Chr={\left\{x_{i}y_{i}\right\}}_{i=1}^{N}$, where $x_{i}\;$ rerpresents the selected features, $y_{i}$ is the categorization of data, and N is the number of samples.$\;x_{i}$ and $y_{i}$ are structured as presented in Equation (10), in order to organize large amounts of EEG information from multiple channels.

(10) $$x_{i}=\left[Ban1_{ch11,ch12,\ldots ,ch1n},Ban2_{ch21,ch22,\ldots ,ch2n},BanX_{chm1,chm2,\ldots ,chmn}\right]\;y_{i}=\left[0|1\right],$$

where $x_{i}\;$ contains the EEG signal’s data following the frequency range order and $y_{i}$ includes the information of two cognitive states. In total, 8210 samples are implemented.

### 2.10. Classifiers

In this investigation, four classifiers were developed to implement the new dataset generated by GALoRIS. The classifiers were designed in three steps, using the algorithms of SVM = [Linear: RBF], LiR, and k-NN. The first step consisted of pre-processing the information, where the data were divided into two groups: Training and testing. Here, 90% of the samples were used to train the model, and 10% were used to perform the tests. The second step consisted of building the model with data destined to train the model. The parameters and configurations of the model were adjusted. The last step was to evaluate the trained model using data dedicated to testing the model.

The information was divided into training and test sets using k-fold cross-validation (k = 10). k-fold is characterized by avoiding the overfitting of data during the model’s construction, being the most frequently used technique in prediction studies [39]. k-fold randomly divides the data into k subsets of an equal size, where the k-1 subset is used during the validation step, and the rest of the subsets are used in the training step. The process is repeated k = 10 times when performance metrics are calculated to evaluate each cycle model. The k results are averaged to obtain a single estimate. The technique’s advantages are that all test sets are independent, and the result’s reliability is improved k times [22,33].

The metrics used to evaluate the performance of the model are the sensitivity and precision. The sensitivity metric evaluates cases that are correctly classified as true and is calculated with predictions made correctly as a low cognitive workload (CLCW) and predictions made incorrectly as a high cognitive workload (IHCW), as shown in Equation (11):

(11) $$sensitivity=\frac{CLCW}{CLCW+IHCW}*100.$$

The accuracy metric is related to the total number of predictions made correctly and is calculated with CLCW, predictions made correctly as a high cognitive workload (CHCW), predictions made incorrectly as a low cognitive workload (ILCW), and IHCW, as shown in Equation (12):

(12) $$Accuracy=\frac{CLCW+CHCW}{CLCW+ILCW+IHCW+CHCW}*100.$$

### 2.11. Label

In the real world, data are not labeled. Therefore, in recent years, labeling indices have been developed, which implement the frequency bands δ, θ, α, β, and γ to identify different states, as shown in Table 1. However, these indices only use some bands and/or channels to evaluate people’s states. 

In this research, a labeling technique was developed to identify low and high cognitive workload levels to categorize EEG information by implementing the generated chromosome.

The labeling technique consists of defining the upper and lower threshold of the dataset, and calculating the sample’s average to obtain a vector. Afterward, the vector’s maximum and minimum values are calculated and divided between the cognitive states, obtaining the interval’s size for each state, as shown in Equation (13):

(13) $$Thr\left(dataset\right)=\frac{maxvalue-minvalue}{cognitive\;states},$$

where maxvalue and minvalue represent the minimum and maximum value of the vector samples, respectively; cognitive states represent the number of states to evaluate; and Thr(set) is the size of the interval by state. The values of each sample are compared, where $sample<Thr_{dwn}=0$ or $sample>Thr_{up}$ = 1. This technique finds the peaks in the timeline defined as moments with a high cognitive workload during the experiment.

## 3. Experimentation and Materials

### 3.1. Design of the Experiment

The Lane Change Test (LCT) version 1.2 simulator was used in the experiment, simulating a vehicle’s most frequent driving conditions [49]. LCT is designed to quantitatively measure the level of degradation of the subject’s performance while driving and performing other secondary tasks [42,43,44].

LCT consists of driving on a three-lane highway with a length of 3000 m, at a maximum speed of 60 km/h. Along the way, instructions are presented that tell participants to change lanes through traffic signs that appear next to the highway every 150 m. The signals are activated when there is 40 m between the vehicle and the sign. The participant must carry out the activity indicated by the sign whilst respecting the traffic rules [50]. The experiment lasted approx. 80 min, divided into three phases:

- Baseline: The participant takes a seat and places the Emotiv EPOC sensor on their head [51]. The subject keeps their eyes closed and is acoustically isolated for 10 min, where the sensor is activated to collect information;
- First Task (Task_1): The participant starts driving the vehicle without any distraction. During driving, the EEG signals, ISA, and ER are collected. In the end, NASA-TLX is applied;
- Second task (Task_2): In order to increase the subject’s cognitive workload levels, the stress induction protocol proposed in [7] is applied as a second task. The task consists of the random mentioning of a series of digits that the participant has to repeat, following the order of the set of numbers given. All measurements are collected.

### 3.2. Subjective Measures

ISA is a questionnaire applied every 2 min during the development of an activity. The participant must provide the number that best describes their stress level, following a scale of 1 to 5: (1) boring; (2) relaxed; (3) comfortable; (4) little busy; and (5) very busy [52]. The questionnaire’s weighting is calculated by assigning a weight ranging from 1 to 10 to each task, according to the level of difficulty of the task, where 1 represents a low difficulty task and 10 is a high difficulty task. The assigned weight is multiplied by the number provided and averaged for the activities to obtain the ISA weighting ranging from 1 to 100. 

NASA-TLX is a post-exercise application that evaluates six factors defined as dimensions that characterize the subjective workload [53]. The methodology proposed in [24] is used to obtain the scale, ranging from 1 to 100. 

### 3.3. Measurement of the Vehicle Performance

The vehicle performance is associated with the ability to keep the vehicle within safety margins. To assess this capacity, ER was implemented in this investigation. ER evaluates the total activities performed incorrectly concerning all of the activities presented during the experiment. In [15], the authors explain the relationship between ER and high levels of cognitive workload. The greater the number of activities carried out during a task, the higher the cognitive workload, increasing the error rate. To estimate the ER of each subject, Equation (14) is defined, where the sum of the activities carried out erroneously ($a_{e}$) in relation to the total activities ($a_{t}$) presented during the task is calculated.

(14) $$ET=\sum_{i=0}^{a_{t}=20}\frac{a_{e}}{a_{t}},$$

where i goes from no error to the maximum number of defined activities, where the activities (a) are the lane changes exhibited during the simulation. The errors occur when the lane changes are not performed. 

### 3.4. Collection and Extraction of EEG Signals

The EEG signal was acquired using the 14-electrode Emotiv EPOC headset sensor. The sensor sent the signal wirelessly to a USB receiver and stored the information in an edk.dll file.

**Figure 5:**
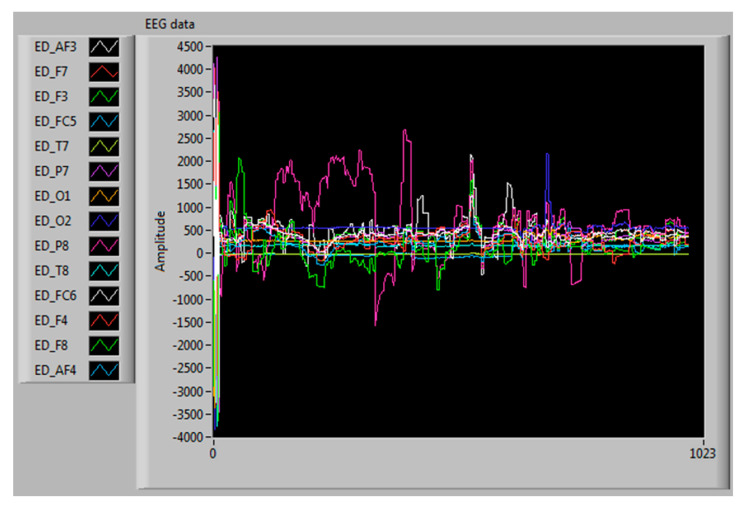
Acquisition of the EEG signal in real-time.

An application was developed with the LabVIEW Instrument using the edk.dll file to analyze and visualize the EEG signal in real-time, as shown in Figure 5. The information was stored in a file with the extension *.cvs, using the microvolt unit of measure. A 16 GB of RAM computer with an Intel Core i7 (2.8 GHz) processor was used.

A feature extraction process was implemented to analyze the collected information. This method consisted of transforming the original signals into a vector of features representing the signal’s behavior. In the literature, features in the time domain, frequency domain, and time-frequency domain are distinguished [54]. In this investigation, the signal was analyzed in the frequency domain using the spectral power density (PSD). PSD determines the distribution of the signal power in a frequency range, facilitating the extraction of the most popular features in the context of the cognitive workload [55]. These features are defined as frequency bands and are Delta (0.5–4 Hz), Theta (4–8 Hz), Alpha (8–12 Hz), Beta (12–30 Hz), and Gamma (30–100 Hz) [23,56,57]. 

The signals are sensitive to activities called artifacts generated by the body’s movement, which alter the quality of the signal [36]. Artifacts were removed by implementing the Butterworth filter of order 5 with a cutoff frequency of 1 to 100 Hz based on [29,51,52]. Butterworth has a greater linear response than other filters, allowing the efficient filtering and decomposition of EEG signals [58].

Fast Fourier Transform (FFT) was calculated with a Hanning window of 128 samples at a length of T = 5s, in order to convert the signal from the time domain to the frequency domain and extract the magnitude of the power spectrum of the delta, theta, alpha, beta, and gamma frequency bands. 

The data format was channel * sample_number * frequency_bands (9 ×  8210 ×  5). All information was standardized.

**Figure 6:**
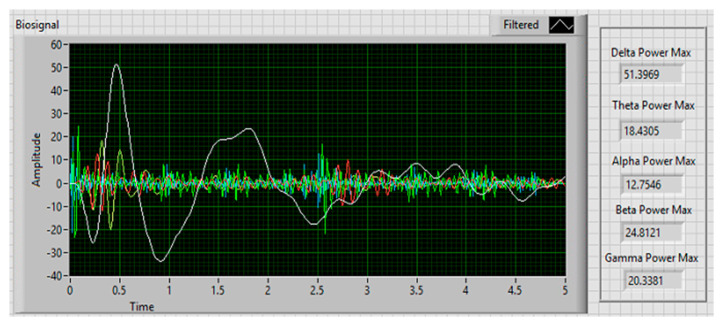
Extraction of the five frequency bands.

An interface was developed using LabVIEW to obtain the EEG data and extract the frequency bands implementing PSD. Figure 6 shows the interface, where the signal frequency distribution extracted from each of the bands can be observed. The maximum value of the power spectrum’s magnitude was stored in a file with the extension *.csv [59]. 

### 3.5. Dataset and Parameters

In [8,44,45], the authors suggest that using a combination of the band’s information helps to identify cognitive states, obtaining better results in the classifier. In this research, seven subsets were built based on four principles to analyze the information’s behavior, the relationship between the features, and the prediction model’s performance, as shown in Table 2.

First, a dataset with all of the data was built to analyze the data. Second, a dataset was constructed with the alpha band’s information characterized by efficiently recognizing cognitive states [60]. Third, a dataset was built with the beta and gamma band information related to a single cognitive state [55,61]. Finally, four datasets were constructed with information related to two cognitive states [36,62]. The band’s information was combined. All datasets followed the criterion of statistical selection, where $B_{ch}\leq a∴\;\in Search\;Space$.

**Figure 7:**
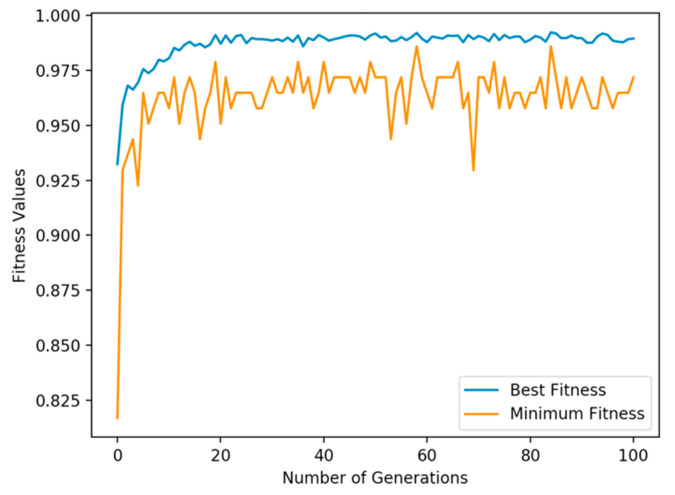
GALoRIS performance analysis evaluating different generations with a population size of 100.

The parameters defined in this work are based on [31,58,63,64] and were configured during model development in the training phase, selecting the one that obtained the best performance. For GALoRIS, the number of generations is 30, with a population size of 100 genes for each generation. A tournament selection of size t = 5 is configured, where individuals are “turned” *t* times to be selected. The two-point crossover is established with a probability of crossing of 0.8 to perform mating between two individuals. The mutation is simple, with a probability of mutating of 0.1. In Figure 7, the analysis of the performance of GALoRIS during the evolutionary process is presented. In particular, with a population of 100, the algorithm achieved the best performance from generation 30.

For SVM, the parameters were C = [0.0001, 1000] and γ= [0.00001, 10], and for k-NN, it was k = [1, 10]. 

GALoRIS was used as a hyperparameter selection strategy for SVMRBF and k-NN. RiL and SVMLineal were implemented with a basic configuration.

## 4. Results

### 4.1. Subjective and Vehicle Performance Measures

The results obtained from ISA, NASA-TLX, and ER in the experiment are presented in Table 3. The results obtained in task_2 were greater than those in task_1 in terms of all measures, where the subjects showed an increase in the cognitive workload during the experiment’s phases. The data of subject_2 were deleted because the subject presented sickness problems during the experiment.

### 4.2. EEG Signals

Table 4 presents a descriptive analysis of each of the frequency bands extracted from the EEG signals. The results show that the values of the alpha, beta, and gamma bands in task_2 were higher than those in task_1. Furthermore, the results of the delta and theta band increased during tarea_1. These results are due to the fact each band is related to a cognitive state [8,59,65,66,67]. For example, the increment in delta [68] or theta [61,69] wave activity is associated with a low cognitive workload, fatigue, or a relaxation state. The increment in alpha [28,70], beta [68], or gamma [65,71] wave activity is associated with a high cognitive workload, stress state, or overload of mental effort.

### 4.3. Statistical Test Results

Table 5 shows the results obtained from the Student t-test, where the mean, standard deviation, and *p*-value of each measure obtained during task_1 and task_2 can be observed. 

The ISA results indicate a significant difference between task_1 (M = 23.6, S = 38.1, t(3) = −11.54) and task_2 (M = 46.6, S = 101.2), with a value of *p* ≤ 0.001. NASA-TLX had a value of *p* ≤ 0.04, where task_1 (M = 25.4, S = 715.7, t(3) = −3.2) and task_2 (M = 65.4, S = 38.2) exhibited significant differences.

ER had a value of *p* ≤ 0.02, where task_1 (M = 3, S = 0.6, t(3) = −3.9) and task_2 (M = 8, S = 8) exhibited significant differences. The values obtained in the EEG signals were alpha (M = −0.20, SD = 0.17), beta (M = −0.085, SD = 0.60), delta (M = 110.2, SD = 0.81), and gamma (M = −0.24, SD = 0.09), with values of (*p* ≤ 0.05, t(4) = −2.656), (*p* ≤ 0.03, t(4) = −3.119), (*p* ≤ 0.03, t(4) = 3.041), and (*p* ≤ 0.005, t(4)= −5.529), presenting statistically differences between the two phases of the experiment, where alpha, beta, and gamma obtained higher values with a high cognitive workload. Moreover, delta obtained higher values with a low cognitive workload. The theta band (M = 0.20, SD = 0.477), with (*p* ≤ 0.383, t(4) = 0.980), did not present a significant difference. 

Table 6 presents the correlation index between the subjective, vehicle performance and EEG signal, where the correlation is generally medium-high. Of the examined measures, ISA and RT presented a medium-high correlation, with alpha (r2 = 0.3, r2 = 0.6), beta (r2 = 0.4, r2 = 0.6), delta (r2 = −0.5, r2 = −0.7), and gamma (r2 = 0.6, r2 = 0.8), suggesting a convergence between these measures. NASA-TLX is an independent measure of physiological measures, as in [72], which may be due to a post-exercise measure. Additionally, the theta band demonstrated independence, with subjective and performance measures.

### 4.4. Labeling Results

The results of applying the data labeling methodology in dataset_1 are $Thr_{up}=\left[0.0076,\;0.0110\right)$ and $Thr_{dwn}=\left[0.0110,\;0.0176\right)$ labeling the data as $Thr_{up}=0$ and $Thr_{dwn}=1$. The interval threshold values for each dataset are dataset_2 = $Thr_{up}=\left[0.0049,\;0.0084\right)$ and $Thr_{dwn}=\left[0.0084,\;0.0113\right)$, Dataset_3 = $Thr_{up}=\left[0.0036,0.0110\right)$ and $Thr_{dwn}=\left[\;0.0110,0.0130\right)$, Dataset_4 = $Thr_{up}=\left[0.0043,0.0110\right)$ and $Thr_{dwn}=\left[\;0.0110,0.0131\right)$, Dataset_5 = $Thr_{up}=\left[0.0072,0.0110\right)$ and $Thr_{dwn}=\left[\;0.0110,0.0134\right)$, Dataset_6 = $Thr_{up}=\left[0.0026,0.0110\right)$ and $Thr_{dwn}=\left[\;0.0110,0.0131\right)$, and Dataset_7 = $\;Thr_{up}=\left[0.0084,0.0110\right)$ and $Thr_{dwn}=\left[\;0.0110,0.0133\right)$. The threshold interval range is *x* < 0.0110 ≤ *x* in most cases. 

### 4.5. GALoRIS Results

Table 7 presents the GALoRIS results, where the AP obtained from each dataset created can be observed. For example, in subset_1, the proposed method reduced the number of attributes from 36 to 13 features on average, representing 64% less of the original data, and obtained a 97% performance for adjustment of the elements. A considerable reduction in the original dataset’s dimensionality generates a more efficient model and is ideal in real-time applications.

Subset_2 achieved a 77% performance, with 3 selected features; subset_3 obtained 88%, with 11 selected features; subset_4 achieved 94%, identifying 16 features with relevant information; in subset_5, 17 features were identified, obtaining 95%; in subset_6, four sets of combinations were defined, with a 96% performance in each with 8, 10, 13, and 18 features; and finally, subset_7 achieved 90%, establishing 19 features.

In addition, Table 7 shows the results of the chromosomes generated in each dataset, where each element of the vector is a chromosomes gene (Chr) that represents whether a feature is selected. For example, in subset_1, the individual created by GALoRIS is Chr=[0, 1, 1, 1, 0, 0, 0, 1, 0, 1, 1, 1, 1, 0, 0, 0, 1, 1, 0, 0, 0, 1, 0, 0, 0, 1, 0, 0, 0, 0, 1, 0, 0, 0, 0, 0], where $x_{n}=1∴\;\in Chr^{\prime }$ is defined as $Chr^{\text{′}}=\left[x_{2},x_{3},x_{4},x_{8},x_{10},x_{11},x_{12},x_{13},x_{17},x_{18},x_{22},x_{26}\right]$. Each element $x_{n}$ corresponds to a feature, creating the new chromosome with 13 selected features, as shown below:

$$Chr^{\text{’}}=[{\mathrm{Delta}}_{\mathrm{AF}4}^{},{\mathrm{Delta}}_{F3}^{},{\mathrm{Delta}}_{F7}^{},{\mathrm{Delta}}_{P8}^{},{\mathrm{Alpha}}_{\mathrm{AF}3}^{},{\mathrm{Alpha}}_{\mathrm{AF}4}^{},{\mathrm{Alpha}}_{F3}^{},{\mathrm{Alpha}}_{F7}^{},{\mathrm{Alpha}}_{P8}^{},{\mathrm{Alpha}}_{T8}^{},{\mathrm{Beta}}_{F7}^{},{\mathrm{Beta}}_{P8}^{},{\mathrm{Gamma}}_{F7}]$$

Table 7 shows that most of the attributes proposed in subset_4 are selected, demonstrating that the combination of alpha and beta bands can identify the vehicle driver’s cognitive states. Otherwise, it can be observed that subse_2 and subse_3 obtained a lower percentage for their performance, with values of 88.79% and 77.34%, respectively.

The features with a high selection rate are Delta_F7 ($p=1.29\times 10^{-27}$), Alpha_AF4 ($p=\;4.14\times 10^{-26}$), Alpha_F3 ($p=5.80\times 10^{-5}$), Alpha_F7 ($p=5.50\times 10^{-16}$), Alpha_F8 ($p=6.03\times 10^{-22}$), Alpha_O2 ($p=9.14\times 10^{-9}$), Alpha_P8 ($p=1.76\times 10^{-15}$), Beta_AF4 ($p=3.38\times 10^{-13}$), and Beta_FC5 ($p=4.19\times 10^{-24}$), demonstrating that they have relevant information that can be used to identify different cognitive states of vehicle drivers. On the other hand, the features with the lowest selection rate are Theta_T8 (*p* = 0.292), Alpha_T8 (*p* = 0.518), Gamma_AF3 (*p* = 0.407), Gamma_AF4 (*p* = 0.501), Gamma_FC5 (*p* = 0.677), Gamma_O2 (*p* = 0.517), and Gamma_T8 (*p* = 0.887).

The GALoRIS’s average runtime is 516.867 s. EEG signals comprise a high dataset dimension [73], and this directly increases the computational complexity by structuring the data, selecting features, and classifying the data.

### 4.6. Classifier Results

Table 8 shows the results obtained with each algorithm. The SVM-RBF obtained, on average, the best performance during the training and testing phases, with a 96.50% and 96.14% accuracy, respectively, and a 96.64% sensitivity in the model, i.e., when the driver is in a specific cognitive state, the model is able to predict that state 96% of the time. k-NN obtained, on average, 95.80%, 95.46%, and 95.47%, respectively. SVM-Linear obtained, on average, 84.97%, 84.87%, and 84.80%, respectively. Finally, LiR achieved, on average, 85.33%, 85.21%, and 85.21%, respectively.

In general, subset_6_1 achieved the best testing performance in the four classifiers, with a 94.68% accuracy on average, followed by subset_4, with a 94.37% accuracy on average; subset_5, which obtained an average of 93.28%; subset_1, with a 93.23% accuracy on average; subset_7, which achieved an average of 92.85%; subset_6_2, with a 92.01% accuracy on average; subset_6_3, which obtained an average of 91.06%; subset_3, with a 90.43% accuracy on average; subset_6_4, with an 88.05% accuracy on average; and finally, subset_2, which achieved an average of 74.23%. Additionally, the table shows the standard deviation obtained in the test phase in each classifier.

## 5. Conclusions and Discussion

The results obtained from GALoRIS were compared with the most frequently used feature selection algorithms in the literature to analyze EEG signals’ Mutual Information (MI) and conduct principal component analysis (PCA) [74]. MI and PCA were evaluated using the seven datasets proposed in this research, and the results are presented in Table 9.

As observed in the table, GALoRIS obtained the best performance results, achieving a total average accuracy in the four classifiers of 90.42%, followed by MI with 83.86% and PCA with 77.81%. GALoRIS-SVMRBF obtained the best results, with a value of 96.14%.

**Figure 8:**
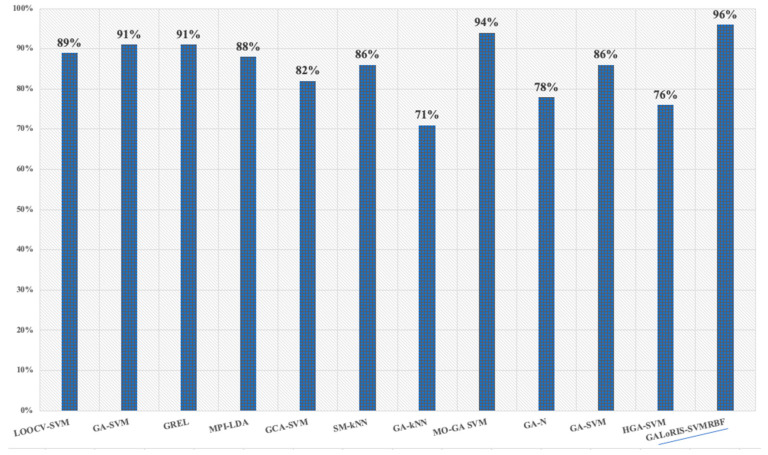
Comparison of the accuracy results obtained from the models related to this work and GALoRIS.

In the literature, work related to this research has been found, as shown in Figure 8. In [75], a feature extraction method was explored based on rhythm entropy to classify the EEG signals. The classification rate achieved was 89.7% using SVM with leave-one-out-cross-validation (LOOCV). In [29], a model with GA and SVM is proposed to classify several databases. The model obtains, on average, a value of 91%. In [76], an algorithm employed to stabilize EEG signal patterns based on a graph regularized extreme learning machine is proposed. It achieved a 69.67% and 91.07% accuracy. In [77], an algorithm for selecting features based on the mutual partial information algorithm that eliminates the less significant information of the EEG signals and develops a classifier using the linear discrimination analysis algorithm is proposed, obtaining an 88.7% accuracy. In [78], the granger causality algorithm is implemented to extract the most relevant EEG signal features and develop a classifier with SVM, obtaining an 82.66% accuracy. In [79], a system for emotion classification based on the EEG signal using statistical measures and KNN is proposed. The system achieved an 86.12% accuracy on average. In [80], emotional stress state detection using a genetic algorithm and k-NN based on EEG signals is proposed. It achieved a 71.76% accuracy. In [81], a system multi-objective genetic algorithm and SVM are designed to find the most relevant features and classify the EEG signal. They achieved a 94.4% accuracy. In [82], feature selection is developed based on a genetic algorithm using regularized neighborhood component analysis to enhance the motor imagery signal’s classification performance. The system achieved a 78.9% accuracy on average. In [83], a classifier based on multimodal EEG data is proposed for depression recognition using genetic algorithms and SVM, k-NN, and decision trees, achieving an accuracy rate of 86.98%. In [84], a feature selection algorithm of EEG oscillatory activity related to motor imagery using a hierarchical genetic algorithm is presented, achieving a 76.04% accuracy. GALoRSI-SVM obtains an accuracy of 96.14% in data classification, significantly improving the classifier performance.

In this study, we have introduced a new feature selection model for pattern recognition called GALoRIS. GALoRIS selects EEG features based on exploring the fusion of information and identifying the principal features that contribute to recognizing cognitive states and structure a new dataset capable of optimizing the classification process to build a robust and powerful learning model. 

The results of this research demonstrate several aspects. First, the measures proposed in this research allow the subject’s level of cognitive workload while driving a vehicle to be evaluated. Second, statistical tests evaluated the relation between measures and cognitive states to observe the subject’s internal behavior and determine whether different cognitive workload levels could be obtained during the experiment. With the statistical results, it could be observed that when the level of difficulties increased, the drivers perceived an increase in the cognitive workload demand, affecting their concentration and increasing the errors. Third, combining features from multiple sources can improve the model; in fact, an improvement in the classification performance from 10% to 20% could be observed compared to using features from a single data source. Finally, the main objective of GALoRIS is to propose a new search strategy for more efficiently exploring the information of EEG signals and identifying the features that can help describe cognitive states while driving a vehicle. The GALoRIS results show that feature selection algorithms for pattern recognition are fundamental to obtaining high percentages of precision in the prediction models. Moreover, GALoRIS was proven to support datasets of various sizes, selecting the attributes with relevant properties, reducing the original dataset by 64% and maximizing the predictive capacity in the prediction models to achieve a 98% accuracy in information classification. The features used in this research work can be considered as the reference point for identifying a high and low cognitive workload of vehicle drivers.

Although the average processing time of GALoRIS was 516.867 s, this is regarded as an average time based on [39,78,83,85]. It is essential to consider that the selection of features is a procedure that is only carried out once and does not affect the model’s test time. It was observed that the processing time was reduced by 80% at this stage.

Future work on this research topic will implement a new dataset to assess the model’s predictive ability developed in this research.

## Figures and Tables

**Table 1:** Indices used to calculate emotional and cognitive states of people using the electroencephalographic (EEG) signal.

References	States	Metrics
[40]	Lateral Index at Stress	$LIS=\frac{Right-Left}{Right+Left}$
[41]	Cognitive-Affective (Frontal Asymmetry)	$FA=In\frac{\alpha \;Right\;AF4}{\alpha \;Left\;F3}$
[42]	Engagement	$\frac{\beta }{\alpha +\theta }$
[43]	Alert/Stress	$\frac{\theta +\alpha }{\beta }$
[44]	Valence	α(FA3)−β(F3)
[44]	Arousal	$\frac{\beta \left(AF3+AF4+F3+F4\right)}{\alpha \left(AF3+AF4+F3+F4\right)}$
[45]	Alzheimer	$Thr_{up}\left(x\right)=avd\left(x\right)+1.5\cdot stdev\left(x\right)$ $Thr_{dwn}\left(x\right)=avd\left(x\right)-1.5\cdot stdev\left(x\right)$
[46]	Event-related desynchronization	$\frac{band\;power\;refence-band\;power\;test}{band\;power\;reference}*100$
[47]	Neuronal activity	$\frac{\beta }{\theta }$
[48]	Load Index	$\frac{\theta }{\alpha }$
[48]	Equanimity	$\frac{B^{2}-\alpha \left(\alpha -\alpha +\theta \right)}{B^{2}+\alpha \left(\alpha +\theta \right)}$

**Table 2:** Datasets analyzed in the model following the four principles to analyze the information’s behavior.

Dataset	Features	No. of Features
Subset_1	Delta_AF4,Delta_T8,Delta_AF3,Delta_F3, Delta_F7, Delta_F8, Delta_FC5, Delta_O2, Delta_P8, Alpha_AF4, Alpha_F3, Alpha_F7, Alpha_F8, Alpha_FC5, Alpha_O2, Alpha_P8, Alpha_T8, Beta_AF3, Beta_AF4, Beta_F3, Beta_F7, Beta_F8, Beta_FC5, Beta_O2, Beta_P8, Beta_T8, Gamma_AF4, Gamma_F3, Gamma_F7, Gamma_F8, Gamma_FC5, Gamma_O2, Gamma_P8, Gamma_T8	36
Subset_2	Alpha_AF4, Alpha_F3, Alpha_F7, Alpha_F8, Alpha_FC5, Alpha_O2, Alpha_P8, Alpha_T8	9
Subset_3	Beta_AF4, Beta_F3, Beta_F7, Beta_F8, Beta_FC5, Beta_O2, Beta_P8, Beta_T8, Gamma_AF4, Gamma_F3, Gamma_F7, Gamma_F8, Gamma_FC5, Gamma_O2, Gamma_P8, Gamma_T8	18
Subset_4	Alpha_AF4, Alpha_F3, Alpha_F7, Alpha_F8, Alpha_FC5, Alpha_O2, Alpha_P8, Alpha_T8, Beta_AF3, Beta_AF4, Beta_F3, Beta_F7, Beta_F8, Beta_FC5, Beta_O2, Beta_P8, Beta_T8,	18
Subset_5	Alpha_AF4, Alpha_F3, Alpha_F7, Alpha_F8, Alpha_FC5, Alpha_O2, Alpha_P8, Alpha_T8, Beta_AF3, Beta_AF4, Beta_F3, Beta_F7, Beta_F8, Beta_FC5, Beta_O2, Beta_P8, Beta_T8, Gamma_AF4, Gamma_F3, Gamma_F7, Gamma_F8, Gamma_FC5, Gamma_O2, Gamma_P8, Gamma_T8	27
Subset_6	Delta_AF4, Delta_T8, Delta_AF3, Delta_F3, Delta_F7, Delta_F8, Delta_FC5, Delta_O2, Delta_P8, Alpha_AF4, Alpha_F3, Alpha_F7, Alpha_F8, Alpha_FC5, Alpha_O2, Alpha_P8, Alpha_T8, Beta_AF3, Beta_AF4, Beta_F3, Beta_F7, Beta_F8, Beta_FC5, Beta_O2, Beta_P8, Beta_T8	27
Subset_7	Delta_AF4, Delta_T8, Delta_AF3, Delta_F3, Delta_F7, Delta_F8, Delta_FC5, Delta_O2, Delta_P8, Alpha_AF4, Alpha_F3, Alpha_F7, Alpha_F8, Alpha_FC5, Alpha_O2, Alpha_P8, Alpha_T8, Gamma_AF4, Gamma_F3, Gamma_F7, Gamma_F8, Gamma_FC5, Gamma_O2, Gamma_P8, Gamma_T8	27

**Table 3:** Instantaneous Self-Assessment (ISA), NASA-Task Load Index (TLX), and error rate (ER) results of the experiment.

	ISA		NASA-TLX		ER	
Subjects	Task_1	Task_2	Task_1	Task_2	Task_1	Task_2
Subject_1	16.66	34.44	4.33	65.67	3	12
Subject_3	31.10	57.77	12.67	56.67	4	7
Subject_4	25.55	51.10	20.33	70.67	3	8
Subject_5	21.10	43.33	64.33	68.67	2	4
Total	23.10	43.32	28.33	61.80	19	34

**Table 4:** Descriptive analysis of EEG signals.

Bands	Task	Mean	Std. Deviation
Delta	Task_1	10.9193	1.20741
	Task_2	9.8171	0.5733
Theta	Task_1	10.2063	0.4682
	Task_2	9.9971	0.11242
Alpha	Task_1	10.4613	0.48171
	Task_2	10.6696	0.46037
Beta	Task_1	22.4447	0.89813
	Task_2	23.2951	0.3818
Gamma	Task_1	15.5624	0.19241
	Task_2	15.8033	0.16196

**Table 5:** Results of Student’s t-test.

	Task_1	Task_2	*p*-Value
	M ± SD	M ± SD	
NASA-TLX	25.41 ± 715.7	65.42 ± 38.25	*p* ≤ 0.048
ISA	23.60 ± 38.18	46.66 ± 101.24	*p* ≤ 0.001
ER	3 ± 0.66	8.25 ± 8.25	*p* ≤ 0.028
DELTA	0.106 ± 0.084	0.028 ± 0.040	*p* ≤ 0.038
THETA	0.056 ± 0.032	0.041 ± 0.007	*p* ≤ 0.383
ALPHA	0.074 ± 0.033	0.088 ± 0.032	*p* ≤ 0.05
BETA	0.917 ± 0.063	0.977 ± 0.026	*p* ≤ 0.036
GAMMA	0.432 ± 0.013	0.449 ± 0.011	*p* ≤ 0.005

**Table 6:** Results of Pearson’s correlation.

	Subjective		Performance	Physiological Measures				
	ISA	NASA	RT	Alpha	Beta	Delta	Gamma	Theta
ISA	---							
NASA	0.598	---						
RT	0.612	0.538	---					
Alpha	0.301	−0.168	0.680	---				
Beta	0.488	−0.113	0.642	0.873	---			
Delta	−0.519	−0.097	−0.745	−0.830	−0.894	---		
Gamma	0.610	0.062	0.815	0.851	0.856	−0.805	---	
Theta	−0.121	0.206	−0.247	−0.592	−0.727	0.768	−0.329	---

**Table 7:** Experimental results of GALoRIS.

Subset	Chromosomes	Features Selection	# Gens	Acc	ER	Time (s)
**Subset 1**	[0,1,1,1,0,0,0,1,0,1,1,1,1,0,0,0,1,1,0,0,0,1,0,0,0,1,0,0,0,0,1,0,0,0,0,0]	‘Delta_AF4′, ‘Delta_F3′, ‘Delta_F7′, ‘Delta_P8′, ‘Alpha_AF3′, ‘Alpha_AF4′, ‘Alpha_F3′, ‘Alpha_F7′, ‘Alpha_P8′, ‘Alpha_T8′, ‘Beta_F7′, ‘Beta_P8′, ‘Gamma_F7′	13	97.7%	2.26%	580.84
**Subset 2**	[0,0,1,0,1,0,1,0,0]	‘Alpha_F3′, ‘Alpha_F8′, ‘Alpha_O2′	3	77.34%	22.6%	201.67
**Subset 3**	[1,1,1,0,1,1,0,1,1,0,1,0,0,1,1,0,0,1]	‘Beta_AF3′, ‘Beta_AF4′, ‘Beta_F3′, ‘Beta_F8′, ‘Beta_FC5′, ‘Beta_P8′, ‘Beta_T8′, ‘Gamma_AF4′, ‘Gamma_F8′, ‘Gamma_FC5′, ‘Gamma_T8′	11	88.7%	11.2%	394.05
**Subset 4**	[1,1,0,1,1,1,1,1,1,1,1,1,1,0,1,1,1,1]	‘Alpha_AF3′, ‘Alpha_AF4′, ‘Alpha_F7′, ‘Alpha_F8′, ‘Alpha_FC5′, ‘Alpha_O2′, ‘Alpha_P8′, ‘Alpha_T8′, ‘Beta_AF3′, ‘Beta_AF4′, ‘Beta_F3′, ‘Beta_F7′, ‘Beta_FC5′, ‘Beta_O2′, ‘Beta_P8′, ‘Beta_T8′	16	94.4%	5.55%	455.52
**Subset 5**	[0,1,1,1,1,1,1,1,1,0,0,0,1,0,1,1,0,1,1,0,1,0,1,0,1,0,1]	‘Alpha_AF4′, ‘Alpha_F3′, ‘Alpha_F7′, ‘Alpha_F8′, ‘Alpha_FC5′, ‘Alpha_O2′, ‘Alpha_P8′, ‘Alpha_T8′, ‘Beta_F7′, ‘Beta_FC5′, ‘Beta_O2′, ‘Beta_T8′, ‘Gamma_AF3′, ‘Gamma_F3′, ‘Gamma_F8′, ‘Gamma_O2′, ‘Gamma_T8′	17	95.4%	4.51%	637.29
**Subset 61**	[1,0,1,1,0,0,1,1,1,0,1,1,1,1,1,0,0,0,1,1,1,1,0,1,1,0,1]	‘Delta_AF3′, ‘Delta_F3′, ‘Delta_F7′, ‘Delta_O2′, ‘Delta_P8′, ‘Delta_T8′, ‘Alpha_AF4′, ‘Alpha_F3′, ‘Alpha_F7′, ‘Alpha_F8′, ‘Alpha_FC5′, ‘Beta_AF3′, ‘Beta_AF4′, ‘Beta_F3′, ‘Beta_F7′, ‘Beta_FC5′, ‘Beta_O2′, ‘Beta_T8′	18	96.5%	3.42%	618.34
**Subset 62**	[1,0,1,1,1,0,1,1,1,0,0,0,0,0,0,1,0,1,0,1,1,0,0,1,0,0,1]	‘Delta_AF3′, ‘Delta_F3′, ‘Delta_F7′, ‘Delta_F8′, ‘Delta_O2′, ‘Delta_P8′, ‘Delta_T8′, ‘Alpha_O2′, ‘Alpha_T8′, ‘Beta_AF4′, ‘Beta_F3′, ‘Beta_FC5′, ‘Beta_T8′	13	96.5%	3.42%	618.34
**Subset 63**	[1,0,0,1,1,0,1,1,0,0,0,0,0,0,1,0,0,0,1,1,0,0,0,1,1,0,0]	‘Delta_AF3′, ‘Delta_F7′, ‘Delta_F8′, ‘Delta_O2′, ‘Delta_P8′, ‘Alpha_FC5′, ‘Beta_AF3′, ‘Beta_AF4′, ‘Beta_FC5′, ‘Beta_O2′	10	96.5%	3.42%	618.34
**Subset 64**	[0,0,0,0,0,0,0,0,1,1,0,0,1,0,0,0,1,1,1,1,0,0,0,0,1,0,0,1]	‘Delta_T8′, ‘Alpha_AF3′, ‘Alpha_F7′, ‘Alpha_P8′, ‘Alpha_T8′, ‘Beta_AF3′, ‘Beta_AF4′, ‘Beta_O2′	8	96.5%	3.42%	618.34
**Subset 7**	[1,1,0,1,1,0,0,0,1,1,1,1,0,1,0,1,1,0,1,1,1,1,1,1,1,0,1]	‘Delta_AF3′, ‘Delta_AF4′, ‘Delta_F7′, ‘Delta_F8′, ‘Delta_T8′, ‘Alpha_AF3′, ‘Alpha_AF4′, ‘Alpha_F3′, ‘Alpha_F8′, ‘Alpha_O2′, ‘Alpha_P8′, ‘Gamma_AF3′, ‘Gamma_AF4′, ‘Gamma_F3′, ‘Gamma_F7′, ‘Gamma_F8′, ‘Gamma_FC5′, ‘Gamma_O2′, ‘Gamma_T8′	19	90.25%	9.75%	425.94

**Table 8:** Classifier results obtained with the linear support vector machine (SVM), SVM-radial basis function (RBF), k-nearest neighbors (k-NN), and linear regression (LiR).

Subset	SVMRBF			k-NN			SVMLINEAL			LiR		
	Train	Test	Sens	Train	Test	Sens	Train	Test	Sens	Train	Test	Sens
Subset 1	96.77	96.71	96.64	97.67	97.50	97.50	89.38	89.29	89.36	89.57	89.43	89.46
Subset 2	85.50	84.36	84.34	82.59	81.66	81.89	66.03	65.97	65.92	65.02	64.96	64.94
Subset 3	97.61	97.02	97.00	94.91	94.26	94.38	85.60	85.57	85.53	85.02	84.87	84.92
Subset 4	98.27	98.16	98.08	98.70	98.50	98.50	91.02	90.73	90.68	90.25	90.09	90.06
Subset 5	97.70	97.27	97.28	97.61	97.46	97.42	89.66	89.50	89.40	89.06	88.91	88.89
Subset 61	98.38	98.24	98.28	98.76	98.64	98.60	91.39	91.27	91.18	90.79	90.59	90.78
Subset 62	96.75	96.54	96.57	98.40	98.17	98.20	86.90	86.86	86.80	86.52	86.47	86.38
Subset 63	98.54	98.27	98.27	97.28	96.90	96.98	84.71	84.64	84.49	84.58	84.45	84.43
Subset 64	97.97	97.72	97.67	95.38	95.03	94.84	79.97	79.96	79.90	79.59	79.50	79.51
Subset 7	97.55	97.17	97.14	96.73	96.50	96.35	85.08	84.94	84.78	92.95	92.82	92.80
Total	96.50	96.14	96.64	95.80	95.46	95.47	84.97	84.87	84.80	85.33	85.21	85.21

**Table 9:** Performance results of the four classifiers using the GALoRIS, Mutual Information (MI), and principal component analysis (PCA) algorithms.

Subset	GALoRIS				MI				PCA			
	SVM RBF	k-NN	SVM	LiR	SVM RBF	k-NN	SVM	LiR	SVM RBF	k-NN	SVM	LiR
Subset 1	96.77	97.50	89.29	89.43	87.78	86.87	76.37	77.40	80.48	80.08	69.03	68.78
Subset 2	84.36	81.66	65.97	64.96	98.78	98.17	98.32	97.65	98.66	99.33	98.62	98.72
Subset 3	97.02	94.26	85.57	84.87	88.00	86.87	76.37	77.40	86.05	85.38	83.46	83.43
Subset 4	98.16	98.50	90.73	90.09	84.65	81.21	78.47	76.85	79.38	78.19	60.44	61.26
Subset 5	97.70	97.46	89.50	88.91	87.78	86.87	76.37	77.40	76.33	75.06	62.39	62.08
Subset 6	97.91	97.18	85.68	85.25	87.08	85.26	78.68	77.43	83.16	82.42	68.17	67.75
Subset 7	97.17	96.50	84.94	92.82	85.53	82.06	76.40	76.12	79.59	79.26	65.89	65.46
Total	96.14	95.46	84.87	85.21	88.51	86.76	80.14	80.04	83.38	82.82	72.57	72.50

## Data Availability

The datasets generated and/or analysed during the current study are available from the corresponding authors on reasonable request.
